# Blood transfusion in elderly patients with chronic anemia: a qualitative analysis of the general practitioners’ attitudes

**DOI:** 10.1186/s12875-017-0647-8

**Published:** 2017-07-11

**Authors:** Sylvain Le Calvé, Dominique Somme, Joaquim Prud’homm, Aline Corvol

**Affiliations:** 1 Unité de Soins de Longue Durée, Centre Hospitalier de Saint Malo, La Briantais, 78, boulevard du rosais, 35400 Saint-Malo, France; 2 CHU de Rennes, Service de gériatrie, Rennes, France; 3 Université Rennes 1, Faculté de Médecine, Rennes, France; 4 Centre de recherche sur l’action politique en Europe, UMR 6051, Rennes, France

**Keywords:** Anemia, Transfusion, Elderly, GP, Decision, Referral

## Abstract

**Background:**

Blood transfusion in chronic anemia is not covered by guidelines specific to older adults. When they consider that this treatment is necessary in elderly patients, French general practitioners (GPs) contact a hospital specialist to plan a transfusion.

**Methods:**

Twenty French GPs were questioned individually regarding their approach to blood transfusion using semi-structured interviews. Each interview was recorded, typed up verbatim and then coded using an inductive procedure by theme, in a cross-over design (two researchers) in two phases: analysis and summary, followed by grouping of the recorded comments.

**Results:**

The criteria for transfusion were hemoglobin level < 8 g/dL and cardiac comorbidities. Some geriatric issues, such as cognitive disorder or dependence, were considered, either as aspects of frailty favoring transfusion or as markers of reduced life expectancy that limit care. Falls and fear of an unpleasant death from anemia prompted GPs to order blood transfusion. The patient’s family provided guidance, but the patient was not routinely consulted. The specialists were rarely asked to participate in decision making. GPs’ perceptions were ambivalent: they considered transfusion to be extraordinary and magical, but also pointless since its effects are transient.

**Conclusion:**

The decision to give a transfusion to an elderly patient with chronic anemia is deemed complex, but GPs seem to take it alone, sometimes guided by the patient’s family. The drawing up of an advance care plan could help involve the patient in decision making.

**Electronic supplementary material:**

The online version of this article (doi:10.1186/s12875-017-0647-8) contains supplementary material, which is available to authorized users.

## Background

### Anemia and transfusion for older patients

Chronic anemia in elderly patients is frequent (affecting >20% of the over-85 s) [1–3] and associated with increased morbidity-mortality [4]. In France, General practitioners (GPs) collaborate with specialists in the follow-up of patients living at home with chronic diseases by prescripting most usual laboratory tests. In the case of chronic anemia, GPs receive results of blood cell counts and can be confronted with a lowering of hemoglobin. So they are called upon to decide whether or not to order a transfusion, which in France can only be given in hospital, mostly in internal or hematology departments. This referral process is peculiar, as GPs don’t expect from the specialist they call (hematologist, internist, or geriatrician) a medical advice; they want him to approve and enact their choice. On his side, the specialist can accept or refuse the transfusion, but will usually organize it after a simple phone call, often in a day hospital, before he can meet the patient.

There are no blood transfusion guidelines specific to chronic anemia in elderly patients [5], and the World Health Organization considers that the definition of anemia is independent of age [6]. However, there is a proven statistical link between anemia and increased cognitive disorders, risk of fall, and hospitalization. Anemia is also associated with reduced muscle strength, decreased bone density, and with reduced quality of life [7].

Several reports indicate that one-third of anemia cases are explained by chronic illness (anemia of chronic disease, including inflammatory diseases and myelodysplasia) and/or by renal insufficiency, one-third by nutritional deficiency (essentially in iron), and one-third are unexplained [4, 5, 7]. As anemia is caused by an underlying illness in two-thirds of cases, the guidelines recommend treatment of the cause. However, symptomatic treatment by blood transfusion may prove necessary if there is no effective etiological treatment or if the anemia is of unknown etiology. Several studies have reported that half of patients receiving blood transfusions are elderly [8–10]. French studies show that blood transfusions have better outcomes in the elderly [11–13], in whom low rates of adverse reactions have been reported [14–16]. Analyses of Medicare databases show increased occurrence of febrile non-hemolytic transfusion reaction and transfusion-related acute lung injury and decreased occurrence of transfusion-associated circulatory overload in the over-80s, compared with patients aged 65–79 [17–19].

### Current guidelines

The official French guidelines on tranfusion [20, 21] are the subject of professional agreement, albeit without a high level of scientific proof. They do not differentiate between the elderly and the general population or between acute and chronic anemia. The guidelines do, however, specify that people aged 80 or over tolerate anemia less well than children and younger adults, that the clinical signs of anemia depend mainly on speed of onset, and that it is the context that matters and not the hemoglobin level [21].

In the United States, the American Association of Blood Banks guidelines [22] concern a hemodynamically stable population, with no mention of any age criterion, and recommend a restrictive transfusion strategy based on a hemoglobin threshold ≤7 g/dL, or ≤8 g/dL in patients with a history of cardiovascular disease. The authors specify, however, that the strength of these guidelines is limited by the small number of clinical trials in some populations and that clinical trials in the elderly are needed [22]. Comparisons of so-called restrictive and liberal strategies have failed to define precise hemoglobin targets in the elderly with chronic anemia [5, 23–25].

The decision to ask for a blood transfusion to an elderly patient with chronic anemia is complex because various factors have to be taken into account and because GPs are uncertain what benefits to expect for a given patient. The decision is based on the patient’s condition and preferences, and on scientific data and the clinical conditions. These factors are included in the decision-making process through clinical expertise, which is central to decision making and which reflects the skill-set and judgment that the clinician has acquired through experience and clinical practice [26]. Clinical expertise incorporates the data of evidence-based medicine and the patient’s preferences and values, so as to assess the problem and work towards a decision [27]. The lack of precise guidelines derived from evidence-based medicine means that a GP’s judgment becomes central.

### Objectives

The aim of this survey was to identify GPs’ attitudes regarding blood transfusion in cases of chronic anemia in older adults, so as to understand how they decide whether or not to contact a transfusion center.

## Methods

The decision to order a transfusion is essentially subjective, so it seemed appropriate to use a qualitative approach, with semi-structured recorded interviews. We gathered a purposive sample of GPs, with heterogeneous age, gender, place of work (rural ou urban) and seniority. A single physician interviewed GPs likely to care for elderly outpatients in the Calvados Department (Normandy) in northwestern France.

### Data collection

Twenty GPs seemed to be a number of participants high enough so the probability of reaching data saturation, ie, that no new property, dimension, or relation will emerge during the analysis, was high. Twenty-nine GPs chosen at professional meetings were telephoned to explain the study aims and procedure and to ask them to participate. Twenty agreed and were recontacted to organize a meeting at their office. Had this saturation not been reached, a new sample would have been recruited [28]. An interview guide (Additional file 1) designed for a 30-min individual interview served as a basis. This semi-structured approach was based on the following themes relevant to older adults (>80 years): definition, consequences and prevalence of chronic anemia, indications, non-indications, and consequences of transfusion. These non-remunerated interviews guaranteed confidentiality and anonymity and were conducted between January and July 2013. They were recorded and typed up verbatim.

### Data analysis

Each interview was coded inductively, based on content, using observed data leading to a hypothesis or model. No a priori hypothesis was applied. Emergent themes, related to the GPs’ attitudes, were sought. Two researchers who are GPs experienced in geriatrics and worked independently to code all interviews by themes and categories, before cross-referencing the results. This coding was done in two stages: first, analysis and summary of statements, interview by interview, and second, organization of statements by target theme. Validation of the survey results confirmed that the statements were reproduced faithfully.

## Results

### Characteristics of the population and interviews

The 20 GPs (13 men, 7 women) interviewed were aged 30 to 65, and worked in rural [9], semi-rural [6], or urban [5] settings. They had more than 15 years (12 GPs), 5 to 15 years [5], or less than 5 years [3] of experience of general medicine. Thirteen of the interviewed GPs worked within 30 km of several transfusion centers. The interviews lasted 16 to 43 min.

The Table 1 summarizes main spontaneous replies to our questions, with the number of occurrence. Two questions do not appear in that table as the replies were too heterogeneous.

**Table 1:** Main replies to the interview guide

Theme of questions	Main replies	Number of occurrence out of 20
Applied transfusion treshold	< 8 g/dL Hb	16
	< 9 g/dL Hb	1
	< 7 g/dL Hb	3
Definition and attitude toward anemia	Same definition and attitude	6
	Different definition and attitude	12
	No answer	2
Apprehended geriatric complications	General complications	20
	Speficic geriatric complications	14
	Falls	10
Influence of cognitive impairments	No influence	6
	Influence	14
Influence of autonomy loss	Fear of autonomy loss or autonomy loss as a treshold	6
	Positif effect of transfusion over anemia	5
Family opinion over transfusion decision-making process	Family opinion in a palliative care context	5
	Family opinion about withholding tranfusion	4
	Family opinion in a cognitive impairment context	6
Image of transfusion	Positive image for GPs	19
	Family satisfaction	4
Improved items thanks to transfusion	General status	15
	Psychomotor status	12
Feared complications of transfusion	Circulatory overload	13
	No fear	5

### Patient-related decision criteria

Most GPs applied an absolute hemoglobin level of 8 g/dL as a transfusion threshold. None of the GPs referred to any specific guidelines. Cardiac comorbidities were considered to increase this threshold. The cause of anemia did not appear as a determining factor in the decision-making process of transfusion. The poor clinical safety of anemia was considered as the most important factor, without consideration of the threshold.

“*If the patient’s clinical condition is poor, I don’t bother with figures […] I always think first of the patient. Because statistics are all very well, but they also make us do stupid things. There’s common sense and there’s experience.*” (GP man 18).

These elements can be found in French or American recommendations [20–22], in fact used without being cited.

Age seems to have paradoxical effect on GPs attitudes: they tolerate a lower hemoglobin concentration before starting lab tests for older patients, but propose transfusion earlier. In other terms, the presence of a moderate anemia will be trivialized, but in case of worsening they will react faster.

Falls strongly motivated GPs to order a transfusion. The GPs apprehended other geriatric complications like loss of autonomy, worsening of cognitive disorders, and orthostatic hypotension. More than the slow and insidious complications of chronic anemia, the events of rapid occurrence trigger the process of requesting a transfusion.

The presence of cognitive impairments makes the situation more complex. GPs freely mentioned cognitive disorders and their influence on decision making. However, the GPs’ comments revealed contradictions regarding how cognitive disorders affect their attitudes. Fear of worsening cognitive disorders linked to anemia leads to earlier transfusion, but cognitive disorders also complicate assessment of the safety of chronic anemia, and the risk of behavioral problems during transfusion tends to reduce requests.

“*It’s true that if a patient with dementia starts pulling all the tubes out…*”(GP man 13).

Overall, GPs’ attitudes varied greatly, ranging from those for whom transfusion is conditional upon preservation of higher functions to those who assert that cognitive disorders do not influence their decision, not to mention those who above all are baffled.

Institutionalization of the patient was spontaneously associated with severe cognitive disorders and dependence, with a twofold effect: it complicates assessment of the impact of anemia and potentially worsens its effects, through loss of residual autonomy.

When cognitive disorders are severe, or loss of autonomy major, or sometimes just because of a very old age, transfusion may appear futile.

“*If the patient is in a wheelchair all day, we’re not really going to see any difference, are we?*” (GP man 14).

The GPs held differing opinions regarding palliative care for an incurable disease. Some favored transfusion at the end of life, while others were in theory opposed, perhaps because they lacked experience. A perceived unpleasantness of death caused by anemia (drawn out, physical and mental discomfort) increased the likelihood that a transfusion would be ordered.

“*I won’t let somebody die of anemia. It would be like letting someone die of hunger*.” (GP man 18).

All GPs were of the opinion that there is no indication for blood transfusion when death is imminent, citing cost and availability. So palliative care situations, as cognitive impairment, very old age or loss of autonomy, can lead to opposite decisions: early transfusions because of patients’ frailty, or transfusion avoidance because this treatment is seen as invasive and useless.

### Attitudes to transfusion

Physicians unanimously see transfusion as “magical”, with “spectacular” effects.

“*We prolong life*.” (GP woman 7).

*“It’s crazy, almost like vitamins, like doping […]Suddenly they wake up!*” (GP man 13).

This excellent image of transfusion seems to be shared with the patients and their families, who retain the immediate effects of this quite simple therapeutic act. One GP recalled a patient with end-stage kidney disease who refused dialysis but agreed to transfusion.

“*He didn’t want to be dialyzed: he already had been once or twice […], but he did want transfusions, because he experienced an immediate effect*” (GP man 13).

The satisfaction of patients, and consequently of their families, is for the physician a token of recognition.

Furthermore, most GPs interviewed considered the risk-benefit ratio as always being favorable, and pointed to psychomotor improvement, as well as improved general condition or mood, and a favorable impact on daily activities. Cognitive improvement in particular was highlighted. No concern was expressed regarding the risk of infection or of incompatibility. The only risks mentioned were volume overload and sometimes delirium.

“*If it bothers a patient to undergo transfusion, if he comes back afterwards highly agitated and with behavioral problems…*”(GP man 14).

Compared with the extraordinary efficacy of transfusion, these risks seem insignificant.

Despite these very positive descriptions of transfusion, GPs frequently expressed unease when taking the decision to recommend a transfusion, as they did not feel sufficiently competent or experienced.

“*I don’t feel comfortable with it […]I haven’t had enough training, or perhaps I haven’t been interested enough, or maybe I haven’t had to face the problem often enough.*” (GP man 16).

The impression of lack of competence is easily understandable since there are no precise recommendations in this type of situation. Moreover, the low frequency of confrontation with this situation does not allow learning by experience. But beyond lack of knowledge and experience, the unease expressed may be linked to ambivalence regarding transfusion, which is seen as extraordinarily effective in the short term, but completely useless in the long term.

“*We make do by filling a vase with a hole in it.*” (GP man 1).

So the patient and/or the patient’s family bestow recognition on the GP, who feels at a loss in the more or less long term. The power of medicine therefore seems illusory if the question of disease progression has not been addressed with the patient.

### A lone decision

GPs faced with decisions regarding transfusions feel that they are responsible for a weighty decision, as they attribute to transfusion the power to prolong or even to restore life. But despite the complexity of the decision, few practitioners reported seeking support of specialists or decision sharing with patients and family. The relation with the specialist who performs the transfusion was mentioned by only two of the 20 GPs interviewed. One GP anticipated a refusal by the specialist, with no possibility of dialogue, above a hemoglobin threshold of 8 g/dL. The other was the only one thinking in terms of a collaborative approach, useful in decision making.

“*I’d propose it jointly with the specialist. We could agree on it together.*” (GP man 16).

It is surprising, in a complex situation where the physician feels uncomfortable and uncertain, that the specialist physician is not contacted either for decision support or for shared responsibility, but rather to confirm the orientation proposed by the general practitioner and to perform the act as a service provider.

Patient’s family circle finally appears as more influent than specialist in decision making. First of all, because the very presence of the family made an order for transfusion more likely. And second, because the GPs involved the family in decision making, notably in complex situations such as transfusion at the end of life and in the case of cognitive disorders.

“*And then afterwards, regarding the family… To know whether or not they wish to prolong their loved one’s life*.” (GP woman 11).

Only one of the GPs interviewed generally preferred not to include the family in decision making.

Interestingly, the patient’s opinion was not explicitly referred to as guiding decision making, and did not seem to be systematically sought, even if some patients expressed weariness after repeated transfusions.

“*There are a lot of patients over 80 who don’t want to be bothered anymore, who don’t want a transfusion.*” (GP woman 12).

## Discussion

### Summary of results

**Fig. 1:**
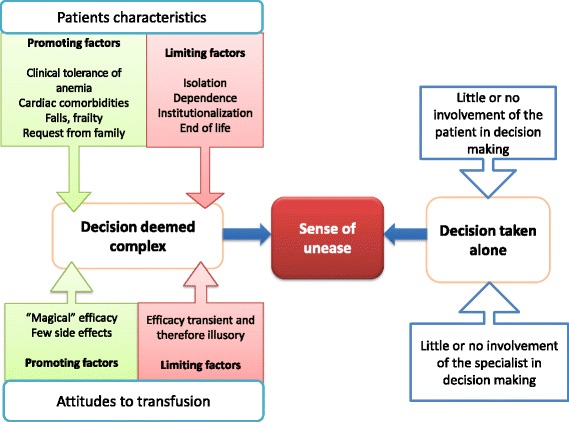
Transfusion

Our results, summarized in Fig. 1, show that GPs are ambivalent regarding blood transfusion, which is seen as life-giving, but also as useless because of the transience of its effects. This creates a sense of unease, which is worsened by GPs’ isolation. One GP apart, they did not refer to the hospital specialist as someone likely to help them in their decision making: they considered that it is up to them to assess the expected benefit for the patient. GPs interviewed were aware of specifically geriatric problems, but were unsure how to include them in a line of reasoning.

The notion of quality of life does not appear explicitly in the decision making process, and patients’ opinions have little place. GPs give thought to the patient’s comfort and to whether the family, which here takes on the mantle of authority, wishes to prolong the patient’s life, but not to what value the patient may attach to his or her current life. More generally, the attitude of French GPs appears paternalistic and lacks consideration for the decisional independence of patients debilitated by age and illness.

### Comparison with previous literature

#### Decision making under uncertainty

Decision making regarding blood transfusion in elderly patients with chronic anemia is clouded by uncertainty, because in a high proportion of patients the etiology of chronic anemia is unknown 4,5,7, and because we lack guidelines specific to such patients [20–22]. Conventionally, in complex situations two ways can be used independently or simultaneously to make a decision [29, 30]: the analytical method, based on scientific data, and the intuitive method, which is based on experience and reasoning by analogy. GPs faced with decisions regarding transfusions in the elderly may experience unease because they cannot base decisions on compelling data or on substantial clinical experience.

A geriatric assessment is clearly valuable when making complex decisions regarding elderly patients, in particular dialysis or the choice of cancer treatment [31–33]. However, our study has showed that GPs currently don’t know how to integrate geriatric factors in transfusion decision making. Furthermore, the question of transfusion in chronic anemia is different from dialysis for exemple because it is not a single important decision involving the long-term therapeutic strategy, but rather lots of “small” decisions, with an approach that in all cases will have to be adapted in light of the patient’s progress. A geriatric assessment could be useful in the process of discussing an advanced care plan, but is usually not feasible at the time of the decision.

#### Shared decision making

The GPs did not feel that their isolation could be lessened by taking the decision together with the patient. It is striking how infrequently the patient’s opinion is taken into account, which raises the question of ageist stereotypes among GPs.

Shared decision making would yet seem realistic and relevant in the context of repeated transfusions: the patient knows the procedure and its associated discomfort, and experiences, or not, its positive effects. Discussion with the patient appears relatively simple, since immediate discomfort must be balanced against the immediate benefit experienced by the patient. The fact that few GPs referred to shared decision making is surprising considering the literature: the absence of strong preference of the medical practitioner considering treatment options and end of life care have been identified as facilitators for shared decision making [34]. If GPs adopted a clearly paternalistic approach, viewing patients as passive and vulnerable, it may be the consequences of GPs’ stereotype and lack of training: the concept of patient-centered care [35] is poorly developed in France [36], and older patients are sometimes deemed incapable of deciding. As in other clinical situations [37, 38], GPs do act according to ageist stereotypes [39]. However, patients factors limiting decision sharing, such as not being empowered or having cognitive limitations [34, 40], have to be considered as well.

Conversely, the families seem to be actively involved in decision-making. It is possible that the participation of the family is sought to share the responsibility for the decision and/or to avoid a conflict in case of disagreement. However, this opposition between patients and family in shared decision should be taken with caution: a study on admission of elderly patients in intensive care units demonstrated that physicians who frequently asked patients about their preferences may be more inclined to asked relative for an opinion [41].

#### GP-specialist relationship

GPs who order a blood transfusion are requesting a procedure that they will neither perform nor are trained to perform. Yet, they don’t seek for specialists’ advice. We can suppose that they regard themselves as the best able to make the decision, acting as “specialist” of patient centered comprehensive care [42]. They consider that transfusion decisions don’t rely on a technical knowledge owned by the specialist, but on their comprehensive approach. Motivation of GPs for collaboration with specialist is known to be largely knowledge driven [43]. It could explain that they don’t seek for collaboration, if they can’t acquire new knowledge this way.

We found in the interviews no elements arguing for a lack of approachability of transfusion specialists. In the absence of economic or organizational constraints, transfusion specialists generally seemed to agree to all requests for transfusion (one exception was related to a hemoglobin level > 8 g), particularly as in the French health care system a blood transfusion is a profitable procedure that takes up little of the specialist’s time.

The interviews did not explicitly address the question of whether the specialist or the GP should explain to the patient the palliative nature of transfusions and involve him in drawing up an advance care plan [44].

### Strengths and limitations

To our knowledge ours is the only survey to have analyzed decisions taken by GPs regarding blood transfusion in elderly patients with chronic anemia. The heterogeneity of the sample of GPs (sex, age, experience, conditions of medical practice) resulted in great variation in their attitudes. A cross-analysis has limited interpretation bias in data slicing and data aggregation. Further studies in different organizational and cultural contexts would be necessary to better describe and understands GPs attitudes.

## Conclusion

Failure to draw up an advance care plan puts the GP in the awkward situation of deciding on an ad hoc basis and prevents any dispassionate discussion with the patient. GPs clearly feel inadequately prepared to address the question of prognosis with their patients and to discuss a treatment plan. The specialist, whether internist, geriatrician, or hematologist, should not act as a simple service provider, but rather should explain the prognosis. A closer collaboration between specialists and GPs would enable the drawing up of an advance care plan, discussed with the patient, and possibly with the family. The GP could refer to such a plan, anticipating the symptoms that should prompt transfusion, when taking decisions in light of disease progression. Such an advance care plan seems essential to facilitate GP-patient communication.

## Additional file

Additional file 1: Interview guide. (DOCX 27 kb)

## Abbreviation

GP: General Practitioner
