# Supplementary material for: Blood transfusion in elderly patients with chronic anemia: a qualitative analysis of the general practitioners’ attitudes
Source: BMC Fam Pract. 2017 Jul 11;18:76. doi: 10.1186/s12875-017-0647-8 (PMC5504771; doi:10.1186/s12875-017-0647-8)
Supplement: Additional file 1: — Interview guide. (DOCX 27 kb) [file 12875_2017_647_MOESM1_ESM.docx]

**Additional files**

**Additional file 1 - Interview guide**

Good morning,

We’re going to address the question of blood transfusion in this survey conducted among 20 general practitioners (GPs) in Calvados. GPs who care for the elderly are the first physicians to receive the results of blood counts performed in patients with chronic anemia. Current recommendations include areas of uncertainty, so we wanted to record and analyze the perceptions and practices of GPs.

Your opinions as a GP are most important. There are, of course, no right or wrong answers. We are interested in your experience and your opinion, even and especially if it is subjective.

For the purposes of this survey, by “elderly” we mean someone over 80 years of age. This is clearly not an absolute cut-off.

We deliberately chose to ask open-ended questions, with the aim of eliciting answers as wide-ranging and free as possible. All answers in this survey will be recorded anonymously and treated in strict confidence.

*→ Definition, consequences*

Q1: How does the definition of anemia differ among the advanced in age?

Q2: What are the consequences of chronic anemia that you fear in the elderly?

*→ Prevalence*

Q3: How many elderly patients with chronic anemia do you see per week, per month?

Q4: What characteristics distinguish the elderly with chronic anemia from the elderly without?

*→ Indications and non-indications for transfusion*

Q5: In what situation do you order a transfusion for an elderly patient?

Q6: In what situation do you never or rarely order a transfusion for an elderly patient (although you consider there is a theoretical indication)?

Q7: In what circumstances, in a home-based patient receiving palliative care, do you order a blood transfusion?

Q8: Does your attitude to blood transfusion change in a patient with cognitive disorders? And according to their severity?

Q9: What difficulties have you already experienced during a blood transfusion in a patient over 80, for whom you considered the indication was clear?

*→ Consequences of transfusion*

Q10: What do you think are the most frequent results after transfusion of a patient over 80 years of age?

Q11: What are the risks of transfusion that you fear?
